# Effect of COVID-19 on dental care for children during the post-pandemic era in Ghana: a concern for policymaking

**DOI:** 10.3389/fdmed.2023.1120685

**Published:** 2023-04-05

**Authors:** Benjamin Adu Agyekum, Lawrence Sena Tuglo

**Affiliations:** ^1^ Department of Community Oral Health and Medicine, College of Health and Well-Being, Kintampo, Ghana; ^2^ Department of Nutrition and Dietetics, School of Allied Health Sciences, University of Health and Allied Sciences, Ho, Ghana

**Keywords:** dental care, children, COVID-19 post-pandemic period, Ghana, increased demand

## Abstract

The impacts of COVID-19 have been felt globally, especially in the delivery of healthcare services such as dental care, although the incidence in Africa is comparatively low. This review article aims to explore the COVID-19 pandemic lockdown and its impact on dental care for children in Ghana. Some dental facilities in Ghana have been experiencing multiple challenges in service delivery, and the ongoing COVID-19 pandemic has aggravated the situation. The COVID-19 pandemic has interrupted all dental care in many parts of Ghana due to its impact on food insecurity, economic breakdown, depression, shortage of essential instrument supplies, inadequate health workers, and poor infection control practices, which present the main risks to dental care. Furthermore, the shutting of dental departments due to the COVID-19 lockdown has restricted the accessibility and utilization of dental services, especially for children. Hence, to avoid further and future pandemics and their impacts on dental care, there is a need to address the implications identified and prepare for the delivery of suitable dental healthcare in Ghana.

## Introduction

The first severe acute respiratory syndrome coronavirus (SARS-CoV) outbreak took place in 2003 in Guangdong, China, and the latest occurred in December 2019 in Wuhan, China (1). The effects of COVID-19 in Africa are comparatively low, and most of the impacts have been heavily felt in low-income countries that already have poor healthcare systems (1). Dentistry is facing a new turning point where dentists working directly in the mouth of the patient contribute to the spread of disease (2). A report published in the United States stated that dentists were at higher risk of contracting COVID-19 from their clients, and while some practices closed, others were operating due to the anxiety of economic fatalities and nonpayment of wages to their staff (1). A study performed in the United Kingdom showed that one-fifth of dentists shut down their services, and more than 70% of the respondents experienced financial loss during the COVID-19 outbreak (1, 3). There are inadequate findings reported in other countries regarding the economic breakdown inflicted on dental services amid the COVID-19 pandemic.

Another concern related to the COVID-19 pandemic is linked to getting dental care in the missed period (4). Throughout dental care, the increased spread of aerosols while drilling and scaling and the physical immediacy between dentists and their clients upsurge the risk of infection (4). The World Health Organization (WHO) has proposed measures during the COVID-19 pandemic to reduce the spread of infection between dentists and clients (5). The WHO prohibited performing aerosol-generating procedures (AGPs) that enable aerosols to be transmitted from one person to another (5). Considering the landscape of dental care and direct contact with saliva, blood, and aerosols from infected persons, dentists are usually at risk of COVID-19 infection. As a result, minimally invasive dentistry (MID) is suggested as an alternative against the pandemic based on the following: understanding the causes of disease and diagnosis; oral healthcare procedures performed by clients and dentists at home and in the hospital, respectively; and the protection of damaged teeth using defensive modalities (5). Hence, this review aims to assess the COVID-19 pandemic lockdown and its impact on dental care for children in Ghana.

## Effect of COVID-19 on dental care

A study by Benahmed et al. (6) showed that only a few of the patients who visited outpatient departments during the pandemic required serious pain management. Marui et al. (7) reported that during the pandemic, most patients reported abscesses and periapical lesions. As reported earlier, patients in need of serious care must be assessed through a phone call rather than coming to the clinic. For an infected person, using an N-95 respirator mask is best for reducing the spread of COVID-19 (8). However, the interruption in dental care might result in more widespread forms of care in the future (1). A person suspected of being infected with COVID-19 must delay visits to dental clinics until the quarantine period is over, and those signs should not be treated as an emergency (1). It has been suggested that good dietary behaviours and active oral sanitation in children prevent oral diseases (9). Hence, complying with a home service routine by brushing the teeth twice daily and the proper use of dental floss and mouth rinse may reduce plaque and prevent the occurrence of oral conditions (10).

## The means of transmission of COVID-19 in dental practice

Infections are transmitted through coughing and sneezing of infected patients and because dental care comprises techniques that produce aerosols (11). The existence of the virus in the saliva of an infected patient can contaminate instruments. The virus can remain on metal and glass surfaces, which healthcare professionals regularly use and are susceptible to infection (12). An experimental study showed that the superficial solidity of COVID-19 in association with SARS revealed that aerosols remained viable for 3 hours, and the COVID-19 virus could persist stably on stainless steel and plastic (13). Additionally, at room temperature, the virus is very contagious on dental instruments and equipment for three days (13).

## Preventive methods of COVID-19 for dentists

It is necessary to implement guidelines for preventing COVID-19. First, patient assessment is compulsory, and each person should be considered a possible case of COVID-19. Television screening should be performed primarily before appointment. For walk-in clients, a non-clinical examination must be implemented according to a standardized COVID-19 questionnaire before starting any clinical process (13). After the dentist is satisfied that a client has no symptoms, dental treatment should be performed using a standardized procedure to avoid faecal-oral virus transmission. Hand hygiene must be practiced before and after the treatment of the patient to avoid contamination (13). Exceptional thought should be given to cutting-edge personal protective equipment (PPE) usage in dental services. These include eyeglasses, face shields, surgical caps, and disposable clothing (13).

Each time clients come for dental service, they should be given mouthwashes of 0.2% hydrogen peroxide, and all care should be performed in a rubber dam to avoid splashes (7). Dental care should comply with strict fumigation rules for cleaning and disinfecting surfaces such as desks, chairs, and door handles and avoid holding any knobs in the dental clinic (14). Appropriate waste disposal should be tailed in bags and coded in a double-layered yellow waste disposal bag (14). Furthermore, as recommended, the use of precise kinds of mouthwash should be dependent on the conditions of the client. A suitable toothbrush should be used for children with erupted teeth. For those without erupted teeth, the gingival mucosa should be washed with sterile gauze dipped in a proper antiseptic solution (6).

## Barriers imposed by COVID-19 on dental care for children in Ghana

During the months that followed the lockdown (May to September 2020), people's health-seeking behaviours changed (15). The number of people reporting to health facilities with other ailments decreased drastically. People were afraid to visit health facilities for fear that they could catch the infection in such places (16). The Director-General of the Ghana Health Service (GHS) advised that all general, nonurgent dental care should be stopped (15). As a result, children in Ghana were deprived of access to dental care checkups, and the eligibility of patients to be seen in regional dental hospitals was regulated. This was to ensure personal protective equipment (PPE) and fallow time requirements, mainly for all aerosol-generating methods. Many relatives stay reasonably anxious about frequently professed “high-risk” environments for nonurgent assessments and management. Despite being a typically preventable disease, dental caries affects 19% of children aged 5 years in Ghana (17) and is a significant health inequity. A recent paper on the indirect effects of COVID-19 emphasized the significant loss of oral health in children (15). This is despite oral health being underlined in the GHS and Ministry of Health (MOH) 2020 Child Health Report.

## How COVID-19 affects Ghanaian communities and dental care for children

In Ghana, the first official cases of COVID-19 were reported on 12 March 2020 (15). As of 6 January 2023, there have been 171,048 confirmed cases of COVID-19 with 1,461 deaths reported to the WHO (18). As of 11 December 2022, a total of 21,400,939 vaccine doses have been administered (18). There are serious effects, such as interference with education, especially among underprivileged children, and the longstanding impact on people and societal fluctuations. Ghana, like other countries, introduced a lockdown that had an impact on children's services, such as abating emergency visits delayed presentation, and missed childhood immunization. Nevertheless, little attention has been given to dental care, a phase of child health that is commonly ignored but must be prioritized (19). In Ghana, over 21,000 children are usually admitted to the hospital due to dental caries each year; denial of this treatment means the additional potential for these children to suffer from pain and infection (17). The country lockdown in Ghana has denied access to routine dental care for children. The disruption of regular care included in the Ghana Health Service and Ministry of Health guidelines has been reserved due to the lockdown. Despite oral health being emphasized as an important healthcare service in pediatric care, the effect of oral health on all phases of life, especially in children, has been denied.

A report by Hewlett et al. (17) in Ghana showed an increase in the admission of dental diseases and related health conditions due to the reduced patronage of oral healthcare during the pandemic, especially in children. From the perspective of dentists, there have been rational anxieties about the financial consequences of the pandemic. Although some health workers were practising, there appeared to have been a universal proclivity to pause elective care and focus on emergencies, which did not bring enough income. The cost of buying enhanced PPE has caused most dental hospitals in major large cities (Hewlett et al. (17); (Kenu et al. (15); Tuglo (20, 21), to shut down during the pandemic. As healthcare workers, an alternative way of getting relatives and advocating the need to reach out to children should be the priority. The dental community was not absent in offering and commenting on their members with official tasks and social duties. In Ghana, dental surgeons have creditably been part of numerous COVID-19 preparedness divisions for diverse clinics and the Ghana Health Service's contact tracing teams (Hewlett et al. (17); (Kenu et al. (15); Tuglo 20, 21). At the global and public level, a directive has been delivered by the WHO, Ghana Health Service, and Ghana Medical and Dental Council. The larger threat to dental care demands extra labour by policymakers to ensure dentists are encouraged and furnished to work without unnecessary risk. It is anticipated that the review will contribute to the understanding of the challenges faced by Ghanaian dentists in service delivery to children during a lockdown, aiding in the design and application of health policies and provision for healthcare workers involved in providing dental services. Although many countries have introduced telephone and video consultations for clients, many diseases have gone undiagnosed and without treatment. Future dental care requirements of the child's family may be anticipated based on the outcome of this review.

## GHS and MOH protocols in comparison to other countries

In England, the United States of America, China and other countries, dentists were instructed to provide an online emergency service, mainly using a telephone triage system, and only refer to crucial care hubs for critical clinical treatment (3, 6, 22, 23). They promote and integrate child oral health in their expert practice by ensuring every contact with high-risk patients is screened and treated before an appointment at the dental clinic to reduce the risk of infection (22). It was done by providing effective mouth care advice for inpatients, proper labelling to local dental services, using oral health resources through a telephone triage system and advocating for the inclusion of oral health within public health policy (3, 22). In Ghana, following the detection that the virus had already started to spread, the government introduced procedures to stop the community's spread of the infection and any additional import of the virus into the country (16). Measures comprised closing all terrestrial borders to the country in addition to the closure of the main international airport in Accra (16). For children in some kindergarten and primary schools, teachers will teach in a classroom, take videos and send them to the children through WhatsApp, while their parents and guardians will pick up their assignments from the schools each week for them to do (16). The GHS also suggested that all open health activities, such as oral health and dental examinations, should be adjourned, which has affected dental care for children (16). Although it helps the Institute as we organize for the expected further COVID-19 waves, these issues should be discussed publicly because of the impact on oral health.

## Remedies to alleviate the burden of dental disease during the pandemic

On 11 March 2020 the World Health Organization declared the COVID-19 outbreak a global pandemic. Vaccines and therapeutic applications are being developed, but there is a long way to go to achieve herd immunity. Traditionally, both patients and clinicians considered that the diagnosis and treatment of dental pain by a dentist in a dental clinic was the best treatment strategy. In the context of the COVID-19 pandemic, the demand for temporary management or minimally invasive treatments has increased (24). With globalization, another pandemic can occur at any time, and the dental ﬁeld must also be prepared for this change. In addition to the pandemic, technological advances, including telemedicine, big data, and artiﬁcial intelligence, have modiﬁed these traditional concepts (25). The most representative home remedies, including tooth brushing and ﬂossing, use of over-the-counter (OTC) medicine, cryotherapy, and traditional remedies with salt and garlic, were selected from the information on how to cope with dental caries. Knowing how to use self-care home remedies to help maintain oral health and reduce dental pain can be a good strategy for responding to this emergency. OTC pain relievers or chlorohexidine gargling, which have antimicrobial and antiviral effects, can provide direct and useful aid in reducing dental pain. The application of cryotherapy to painful or swollen areas is helpful. However, long-term, large-scale studies on the appropriate application time, application dose, side effects, indications, and contraindications of these treatments are lacking (25). Knowing and investigating the appropriate home remedies for dental pain may become more important during the COVID-19 pandemic for immediate pain relief (26). Gargling or tooth brushing with salt is an accessible, cost-effective home remedy that has been used since ancient times in the West and East to reduce pain (26). The important weakness of these traditional home remedies is that, unlike Western medicine, their indications, contraindications, preparation method, action mechanisms, appropriate doses, beneﬁcial effects, risks, and side effects have not been scientiﬁcally proven, and the application method has not been standardized or generalized.

## Conclusion

The shutdown of routine dental care resulted in increased demand for dental care for children during the COVID-19 post-pandemic period. Hence, given the likely post-pandemic increase in care, future preparation and suitable directives for dental care should be prioritized to improve children's oral health and quality of life. This review concluded that the lockdown amid the COVID-19 pandemic had a significant impact on dental care for children in Ghana.

## Recommendations

### Government

The Ghana Health Service and Ministry of Health should designate preventive measures with a discussion with dental authorities. Additionally, to improve the oral health of children, knowledge and behaviours, community programmes should be considered and implemented. Particular attention should be given to the involvement of other healthcare professionals, such as community oral health officers, and educators, such as health promotion officers and parents. As a result, health promotion goes beyond medical treatment and puts health on the agenda of policymakers in all fields and at all levels, directing them to recognize the health implications of their actions and take ownership of their role in maintaining health.

### Educational body

In response to the pandemic, the Ministry of Education and the Ghana Education Service (GES), the agency responsible for implementing policies to ensure that school-age Ghanaians receive education, introduced remote and distance learning programs, including the distribution of learning packages to school children. This response faced the additional challenge of variable access to communications technologies across geographic and socioeconomic status, particularly impacting children in poor and rural households. To prevent learning loss, the International Development Association (IDA) funded the Ghana Accountability for Learning Outcomes Project (GALOP). It supported the Government of Ghana in delivering remote education to an estimated 4.45 million students. The GALOP supported Ghana's COVID-19 Coordinated Education Response Plan during the extended school closure, ensuring the sanitation and safety of schools while also working to build a resilient education system. The project launched distance learning via TV, radio, and online channels. It supported the distribution of printed materials to support home learning. In addition, it provided community-based, complementary basic education classes. The project also helped to launch the Edmodo Learning Management System, which created a platform that enabled all schools, students, and teachers to connect remotely.

### Parents

Parental behaviour is a strong predictor of children's oral health, and parents should participate in oral health education activities aimed at reducing risk factors for acquiring oral disorders. For social, economic, and personal development, good health is a vital asset that must be implemented by parents. The most economical method to enhance oral health among children and, subsequently, their quality of life is by educating parents and encouraging them to promote health in the social and cultural contexts that are best for their children.

### Teachers

Furthermore, to support remedial and accelerated learning that accompanied the reopening of schools in early 2021, the GALOP project supported in-service teacher training for targeted instruction and rapid student assessment to over 70,000 teachers (41 percent of whom are women) in 10,000 beneficiary schools. As the school shutdown was drawing to a close, the project supported back-to-school campaigns. These included radio and TV messages by education managers and practitioners in various languages, encouraging students to return to school.

### Dental council

Every dental clinic and setting should maintain strict booking policies for both elective and nonelective operations to prevent the dental outpatient department area from becoming too crowded. Additionally, without conducting necessary testing, dentists and dental staff should not treat patients who have respiratory problems or fevers. Again, all dental professionals must undergo screening and wear face masks at all times with all books and brochures properly cleaned out, and waiting spaces, floors, tables, handles, and other surfaces must undergo regular disinfection.

### Paediatricians and general practitioners

All the various types of practical disinfectants that are available in Ghana should be distributed so that practitioners can make an informed decision and prevent confusion. Additionally, the dental area should have screening stations for all patients where an evaluation questionnaire is filled out and an infrared thermometer is used to take the patient's temperature until more sensitive screening equipment is available. Officers in charge of screening and triage should wear the proper PPE and follow all established procedures. Seats in OPD and waiting rooms must adhere to social/physical separation guidelines of at least 1.5 meters apart. Streamline the flow of patients and employees through the use of designated entrance and departure locations.

### Surgical area

It is advised that dental assistants and operating dentists must all be completely protected. The N-95 mask, face shield, robe, boots, and hair protection are all included. Additionally, dental surgeons and assistants must wear single-use scrubs. In the clinics, designated areas for putting on and doffing should be present, and a well-organized sterilization system for reusable gowns should be provided. Ahead of receiving treatment, patients should be prompted to gargle for one minute with diluted H_2_O_2_ 1.5% and/or for two minutes with 2% chlorhexidine before the actual procedure is done and, while performing restorative procedures, employ rubber dams as much as feasible without cutting corners. Hydrogen peroxide solution should be used to rinse the teeth before making any access cuts. It is suggested to try and reduce the use of aerosol-producing techniques, such as the use of ultrasonic scalers, air polishing, air/water syringes, tooth prep with an air turbine, or air abrasion, to the absolute minimum notwithstanding all precautions and guidelines.
